# The roles of p38 MAPK → COX2 and NF-κB → COX2 signal pathways in age-related testosterone reduction

**DOI:** 10.1038/s41598-019-46794-5

**Published:** 2019-07-22

**Authors:** Yu Zhao, Xuehui Liu, Yine Qu, Lixuan Wang, Dan Geng, Wei Chen, Li Li, Yangyang Tian, Shiyang Chang, Chunfang Zhao, Xiujun Zhao, Pin Lv

**Affiliations:** 1 Department of Histology and Embryology, Hebei Medical University, Shijiazhuang, 050017 China; 2 Department of Occupational and Environmental Health, Hebei Province Key Laboratory of Environment and Human Health, Hebei Medical University, Shijiazhuang, 050017 China; 3 Department of Histology and Embryology, School of Basic Medical Sciences, North China University of Science and Technology, 063210 Hebei Province, China; 4 Department of human anatomy, Hebei Medical University, Shijiazhuang, 050017 China; 5 Department of cell biology, Hebei Medical University, Shijiazhuang, 050017 China

**Keywords:** Stress signalling, Senescence, Post-translational modifications

## Abstract

In our study, we explored changes in the redox status and inflammatory response in the testes of the SAMP8 model of varying ages (2, 4, 8, 10 months old) compared with control mice SAMR1 by the methods of immunohistochemical staining, Western blotting, RT-PCR and Luminex multi-analyte cytokine profiling. We found that as ROS and inflammation levels increased during aging, steroidogenic enzymes (StAR and P450scc) reduced and led to the decline of testosterone production eventually. The pathways of P38 MAPK → COX2 and NF-κB → COX2 were detected by using specific inhibitors of SB203580 and Bay 11-7082 in isolated Leydig cells. These results indicated that activation of both p38 MAPK → COX2 and NF-κB → COX2 signaling pathways are functionally linked to the oxidative stress response and chronic inflammation during aging, and mediate their inhibitory effects on testosterone production.

## Introduction

Male aging is usually accompanied with decrease in serum testosterone concentration, which are associated with osteoporosis, metabolic syndrome, depression and sexual dysfunction. Although testosterone-replacement therapy can be used, some undesired side effects occur such as increased risk of cardiovascular diseases, prostate cancer, luteinizing hormone (LH) suppression and obesity^1,2^. Therefore, it is necessary to thoroughly understand the mechanism by which steroidogenic function decreases with aging and further develop therapies for hypogonadism.

Testosterone is secreted primarily by testicular Leydig cells and then released into the circulation^3^. The serum testosterone level is known to be affected by a variety of physiological and biochemical factors related with aging^4^. Studies over the past several years showed that ROS level increasing with aging in Leydig cells, particularly lipid peroxidation-mediated damage to cell membrane, is related with intracellular cholesterol transport, which may weaken the function of Leydig cells leading to decreased steroidogenesis^5,6^. It is also known that inflammatory events are involved in the decline of physiological functions of aging organs^7^. Compared to the young, two- to four-fold increments in the levels of blood pro-inflammatory cytokines, such as interleukin-1β (IL-1β), tumor necrosis factor-α (TNF-α), and interleukin-6 (IL-6), are typical in the elderly^3^. It has been shown that TNF-α and IL-1β have an inhibitory effect on the expression of steroidogenic enzymes in Leydig cells^4^.

Although the mechanism by which excessive oxidative stress leads to age-related loss of steroidogenesis is unclear, evidence accumulated in other systems now indicates sustained low levels of oxidative stress during aging can activate the mitogen-activated protein kinase (MAPK) pathway, leading to changes in gene expression that may influence cellular biological reactions and metabolic processes^5,8,9^. Recent reports showed that oxidative damage-associated p38 MAPK signaling pathway was activated in aged rat adrenal cells and Leydig cells. Interestingly, p38 MAPK activity blocked by pharmacological inhibitor in aged adrenal cells led to decrease in phosphorylation of p38 MAPK and increase in cellular steroid synthesis^10,11^. This finding supports the possibility of the role of ROS and p38 MAPK in the reduction of steroid production in elderly steroidogenic cells.

Cyclooxygenase-2 (COX2), an isoform of cyclooxygenase, is a prostanoids enzyme that is responsible for synthesis of thromboxane and prostaglandins. According to the cell type, the expression of COX2 can be induced by cytokines, bacterial endotoxin lipopolysaccharide(LPS) and the tumor promoter phorbol myristate acetate (PMA)^12^. *Cox2* gene in aged Leydig cells is expressed at higher levels, and has been shown to inhibit the expression of steroidogenic acute regulatory protein (StAR)^13^. Further studies showed that COX2 could also be induced by oxidative stress, a process depending on p38 MAPK signaling^14^. There is no clear evidence that elevating oxidative products in aged Leydig cells may inhibit *StAR* expression and steroid production by p38 MAPK activation and higher levels of COX2. In our study, Leydig cells were used to verify the effect of p38 MAPK → COX2 pathway on testosterone synthesis.

NF-κB is an important transcription factor involved in immune reaction, inflammatory diseases and development^15,16^. NF-κB can be activated and transferred from the cytoplasm to the nucleus where it is combined with the promoter regions of several genes, including Cox2, TNF-α and IL-1β^17^. Our study indicated that NF-κB expressed in the nucleus of testis increases with age, but it is not clear whether NF-κB has an effect on testosterone reduction in age-related Leydig cells of by regulating *Cox*2 expression.

In our study, we observed the age-associated changes in testosterone production, the expression of cholesterol side-chain cleavage enzyme (P450scc) and StAR, which are two key enzymes in testosterone synthesis, and investigated the age-related alterations in COX2, IL-1β and TNF-α in 2, 4, 8, and 10-month-old male mice. The results demonstrated that oxidative stress and chronic inflammation are involved in the decline of testosterone production both *in vivo* and *in vitro* with aging. Furthermore, we provide evidence that NF-κB → COX2 and p38 MAPK → COX2 signaling pathways are functionally linked to the oxidative stress response and chronic inflammation during aging and mediates their inhibitory effects on testosterone production. We concluded that both the p38 MAPK → COX2 and NF-κB → COX2 signal pathways played roles in the testosterone reduction with age change.

## Material and Methods

### Animals

Male senescence-accelerated mouse prone 8 (SAMP8,P8) and senescence-resistant inbred strain (SAMR1, R1) mice at 2, 4, 8 and 10 months of age were provided by Hebei Medical University (The Animal approval number is SCXK2014-0004, n = 6 mice per age group). All mice were kept under controlled environment (24–28 °C, 65 ± 5% relative humidity, 12 hour light/12 hour dark cycle) and fed with food and water ad libitum. All animals were handled according to Beijing Laboratory Animal Center’s guidelines, and all programme were appraised and allowed by the Ethics Committee of Hebei Medical University.

### Tissue collection and preparation

All mice were anesthetized with an intraperitoneal (i.p.) injection of chloral hydrate (400 mg/kg body weight). Testes were collected and prepared as my previous paper described^3^. Briefly, the fixed samples were submitted to immunohistochemical staining procedures. Each frozen sample was submitted to protein detection by Western blotting, RT-PCR and Luminex multi-analyte cytokine profiling.

### Isolation of primary Leydig cells

Mice were killed by cervical dislocation, and the testes were aseptically removed and the adipose tissue was dissected. Testes were decapsulated, and then were dissociated with collagenase 1 (500 µg mL^−1^) at 34 °C, with low-speed shaking (90 cycles/min) for 5–10 min until the seminiferous tubules were isolated but retained their structural integrity. Two volumes of DME:F12 (1:1) was added to stop the digestion. Seminiferous tubules were filtered and removed by a 100-µm pore size nylon mesh, then the dissociated cells were collected from the filtrate, centrifuged (15 min, 1500 rpm) and then subjected to centrifugal washing (5 min, 1000 rpm). The final pellet that collected to the 1.0 × 10^6^/mL density fraction was cultured in DME:F12 (1:1) containing 10% FBS, 100 U mL^−1^ streptomycin and 100 U mL^−1^ penicillin, followed by incubation at 37 °C with 5% CO_2_. After 2 h of culture, the medium was displaced with fresh medium after three washes using PBS. Leydig cells were obtained using the differential adhesion speed method. The viability of Leydig cell preparations was detected by incubating with 0.4% Trypan blue for 5 minutes. The viability was 96.5–97%. The purity of cell preparations was evaluated by measuring the percentage of cells stained by immunohistochemical staining of 3β-hydroxysteroid dehydrogenase (3β-HSD)^18^. The >95% purity was achieved. These cell preparations were used to measure the secretion of steroid.

The production of testosterone was measured as previously described^5,19^. Briefly, Leydig cells from young (4 month age) and old (8–10 month age) mice were incubated in the absence (basal) or in the presence of the maximum dose of LH (100 ng/ml) for 2 h. Subsequently, media of incubation were collected and cryopreserved until testosterone production was analyzed by the radioimmunoassay (RIA) technique.

To assess the roles of p38 MAPKs and/or NF-κB inhibitor in regulating steroidogenesis induced by LH in Leydig cells of aged mice, we used the specific p38 MAP kinase inhibitor SB203580 (group SB) and the NF-κB inhibitor Bay 11–7082 either separately (Bay group) or in a specific combination (SB + Bay group). To assess the concentration and the incubation times of Bay as previously described^17^, old Leydig cells were treated with 0–10 μM of Bay for 12 h and incubated with 5 μM Bay for the different time periods (from 0 to 24 h). The concentration and the incubation times of Bay depended on cell viability and the inhibition on the NF-κB expression. The concentration and the incubation times of SB based on a previously published protocol^10^. Triplicate old Leydig cells were pretreated with vehicle alone (control), SB203580 (10 µM) for 1 h, 5 μM Bay 11-7082 for 12 h or SB203580 (10 µM) for 1 h, followed by 5 μM Bay 11-7082 for 12 h. The incubation continued for 2 hours after the addition of LH (100 ng/ml). The medium were collected, frozen and cryopreserved until testosterone levels were analyzed as described above.

### LH and testosterone measurements

Serum testosterone and LH concentrations were evaluated as my last paper described^3^. Briefly, serum testosterone and LH concentrations from individual mice and aliquots of supernatants from testicular homogenates were evaluated using RIA. The sensitivity and intra-assay and inter-assay coefficients of variation of RIA were 13 pg/tube, 8.9%, and 13.6%, respectively.

### Cytokine measurements

xMAP technology (Austin, TX, USA) was used to measure cytokine levels as my last paper described^3^. Briefly, bead sets were coated with capture antibodies for TNF-α and IL-1β. The two cytokines in the samples were recognized by differences in bead sets with fluoregenic emission detection using flow cytometric analysis (Niu and Ro, 2011).

### Measurement of intracellular ROS

The level of ROS was analyzed by measuring the fluorescence of DCFH-DA as as previously described^20^. Briefly, the relative levels of fluorescence were measured with a flow cytometer (Becton-Dickinson, Franklin Lakes, NJ).

### mRNA analysis by semi-quantitative reverse transcriptase or real-time PCR

Total RNA was extracted from treated cells using Trizol reagent (Invitrogen, Carlsbad, CA, USA). Total RNA was frozen at −70 °C until use. Total RNA (2 mg) was reverse transcribed into cDNA by using the SuperScript First-Strand Synthesis System (Invitrogen, Carlsbad, CA, USA). The mRNA was analyzed by semi-quantitative reverse transcriptase as my last paper described^3^. The primer sequences are listed in Supplement 1.

Quantification of mRNA was performed using real-time PCR according to the manufacturer’s instructions for SYBR Premix Ex Taq using a real-time thermal cycler (Bio-Rad, Hercules, CA, USA). Results were expressed as optimal density ratios to *β-actin*. Gene-specific primers used are listed in Supplement 1.

### Western blotting

The protein of COX2, p-p38, p38, StAR, NF-κB was detected by Western blot as last paper described^3^. Briefly, the frozen testes and collected cells were homogenized in lysis buffer. NF-κB nuclear localization experiments refered to manufacturer’s protocol(Thermo Scientific, Pierce Protein Biology Products, Rockford, IL, USA). Each protein sample (50 µg) was separated by SDS-PAGE on different gels (10% gel for NF-κB, COX2, p-p38, p38 or 12% gel for StAR) and then transferred to nitrocellulose membranes. The membranes were incubated with antibodies against NF-κB (Santa Cruz, CA, USA, 1:1000), COX2 (Epitomics Burlingame, CA, USA, 1:1000), p-p38 (Abcam, Cambridge, MA, USA, 1:1000), p38 (Abcam, Cambridge, MA, USA, 1:1000) and StAR (Santa Cruz, CA,USA, 1:1000). Immunoreactive proteins were detected and quantified. To ensure even loading of the samples, the same membrane was probed with β-actin (Santa Cruz, CA, USA,1:5000) or H3 (Santa Cruz, CA, USA, 1:1000) antibody.

### Data analysis

All data were analyzed using SPSS 16, and results expressed as mean ± standard deviation (SD). For all statistical analyses, value of *P* < 0.05 was considered significant. Data were analyzed using two-way ANOVA with complete random design, and then multiple pair-wise comparison was performed using Student-Newman-Keuls *post hoc* tests.

## Results

### Age-dependent changes in LH and testosterone

**Figure 1:**
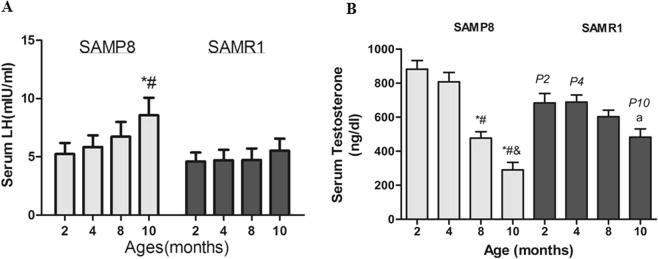
Age and strain-associated changes in serum LH and testosterone. (**A**) Serum LH across P8 mice of 2, 4, 8 and 10 months of age yielded age-associated elevation. (**B**) P8 mice showed a significant decrease in serum testosterone levels with aging, whereas R mice did not. *indicates a significant difference from the mean of the 2-month-old P8 at *P* < 0.05. ^#^*P* < 0.05 vs. 4-month-old P8; ^&^*P* < 0.05 vs. 8-month-old P8; a *P* < 0.05 vs. 2-month-old R1; p2 *P* < 0.05 vs. 2-month-old P8; p4 *P* < 0.05 vs. 4-month-old P8; p10 *P* < 0.05 vs. 10-month-old P8. Each bar represents the mean ± SD.

Experiments were conducted to observe the age-related changes of LH concentration in P8 and R1 mice in different ages (Fig. 1A). A two-way ANOVA was conducted on serum LH levels for the mouse strains (P8, RI) by age (2, 4, 8, 10 months old). The results indicated that age and strain had a significant effect (*P* < 0.01) (Fig. 1A). The interaction of age and strain was significant (*P* < 0.01). A one-way ANOVA conducted on serum LH across P8 mice of 2, 4, and 8 months of age yielded increase with aging but no significant effect (Fig. 1A). The LH levels for 10-month-old was higher compared with 2, 4-month-old in P8 mice (*P* < 0.01), whereas LH levels for 2, 4, 8 and 10-month-old had no significant difference in RI mice.

Experiments were conducted to observe the age-related changes of testosterone production in P8 and R1 mice (Fig. 1B). A two-way ANOVA was conducted on serum testosterone levels for the mouse strains (P8, RI) by age (2, 4, 8, 10 months old). The results showed that age had a significant impact (*P* < 0.01), and that the strain did not (*P* > 0.05) (Fig. 1B). Our results were basically consistent with the results of the previous research^21^. The interaction of strain and age was significant (*P* < 0.01). The testosterone value of the 8-month-old was significantly lower than that of the 4-month-old in P8 mice (P < 0.01), while the testosterone value of the 4 and 8-month- old RI mice had no significant difference. One-way ANOVA of plasma testosterone in 2, 4, 8 and 10-month-old P8 mice produced a significant effect, with testosterone values declining with age (Fig. 1B). The testosterone level of the 4-month-old increased compared with those at 8 or 10 months in P8 mice (P < 0.01) (Fig. 1B). Testosterone level of 8-month-old were reduced by ~44% compared to 4-month-old in P8 mice.

### Age-dependent expression changes in StAR and Cyp11a1 mRNA and protein in testis samples

**Figure 2:**
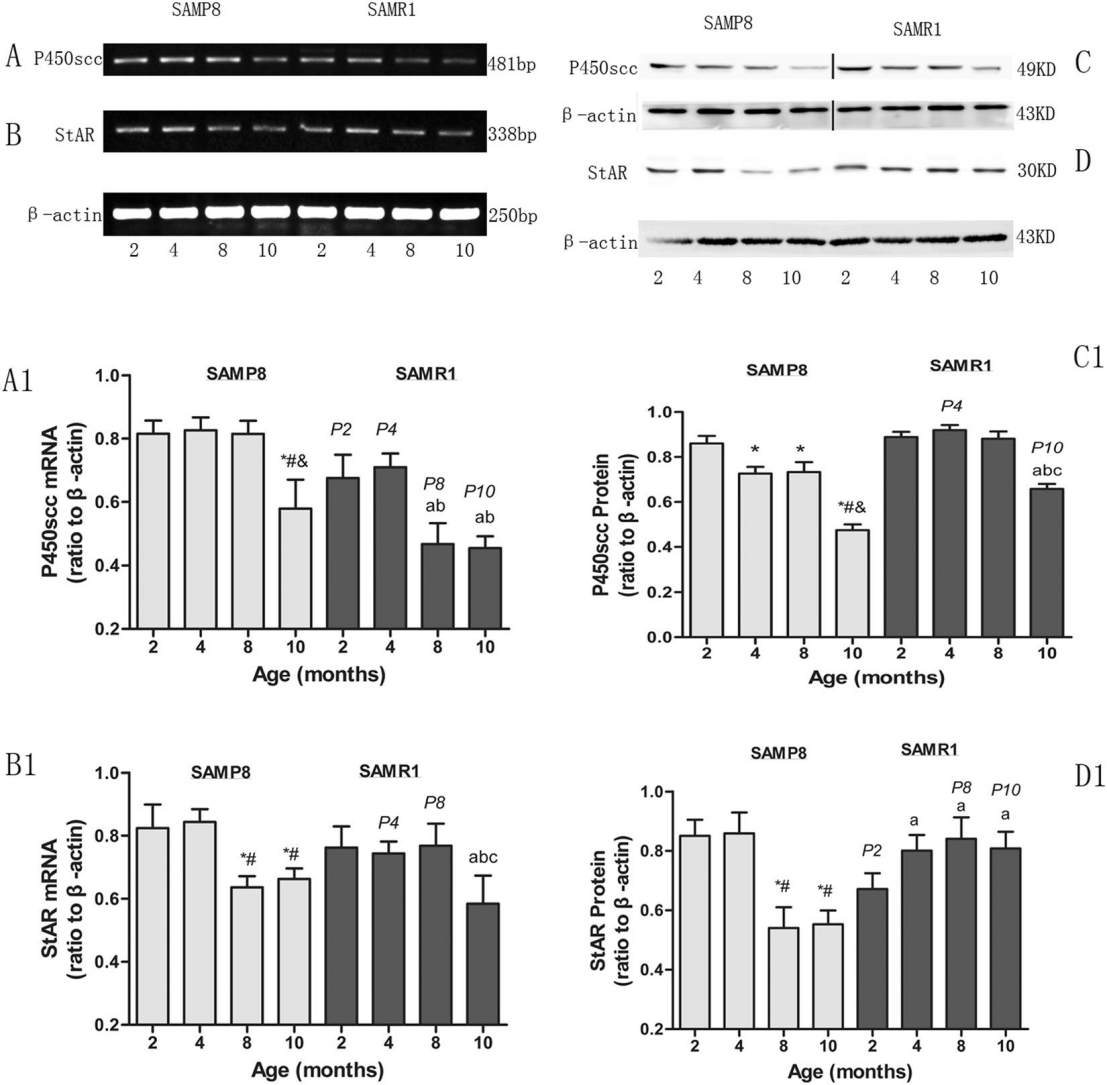
Strain and age-associated alterations in the expression of StAR and Cyp11a1 mRNA and protein. P8 and R1 age groups of 2, 4, 8 and 10 months. (**A**) Cyp11a1 mRNA expression by RT-PCR; (**B**) *StAR* mRNA expression by RT-PCR; (**C**) Cyp11a1 protein expression by Western blot; (**D**) StAR protein expression by Western blot; (A1-D1) Significant difference in strain and age (*P* < 0.05). *P < 0.05 vs. 2-month-old P8; ^#^*P* < 0.05 vs. 4-month-old P8; ^&^*P* < 0.05 vs. 8-month-old P8; a *P* < 0.05 vs. 2-month-old R1; b *P* < 0.05 vs. 4-month-old R1; c *P* < 0.05 vs. 8-month-old R1; p2 *P* < 0.05 vs. 2-month-old P8; p4 *P* < 0.05 vs. 4-month-old P8; p8 *P* < 0.05 vs. 8-month-old P8; p10 *P* < 0.05 vs. 10-month-old P8. Each bar represents the mean ± SD.

A two-way ANOVA was used on the expression of StAR and Cyp11a1 for strain (P8, RI) by age (2, 4, 8, 10 months old) (Fig. 2A–D). The analysis of the StAR expression showed that the interaction of strain and age was significant. However, the interaction of strain and age have no significant impact on Cyp11a1 expression. In P8 mice across age groups of 2, 4, 8, and 10 months, a significant effect was seen in the StAR and Cyp11a1 expression, which decreased with age (Fig. 2A1–D1). The expression level of StAR in 8-month-old decreased compared with that of 4-month-old in P8 mice (*P* < 0.05), however there was no significant difference between the 4-month-old and 8-month-old in RI mice. The expression of Cyp11a1 had no differences between 4-and 8-month-old in P8 mice. The levels of StAR were significantly higher at 2 and 4 months, and they were significantly lower at 8 and 10 months in P8 mice than that of R1 mice (*P* < 0.05). The levels of P450scc at the mRNA level was not consistent with that at the protein level. However, the levels of P450scc at 10 months of age declined compared with those of 2, 4 and 8-month-old mice in P8 and R1 mice. (*P* < 0.01). The levels of P450scc protein were significantly lower at 10 months in P8 mice compared with that of R1 mice.

The P8 mice is characterized by a rapid decline of testosterone at 8 months of age. In order to identify the reasons for this decrease, we detected the change in expression of StAR and Cyp11a1 in testes of P8 mice with age. The level of StAR of 8-month-old mice was significantly lower than those of 2- and 4-month-old mice in P8 mice (*P* < 0.01). This result was consistent with changes in testosterone.

### Age-related alterations in pro-inflammatory cytokines in testis samples

**Figure 3:**
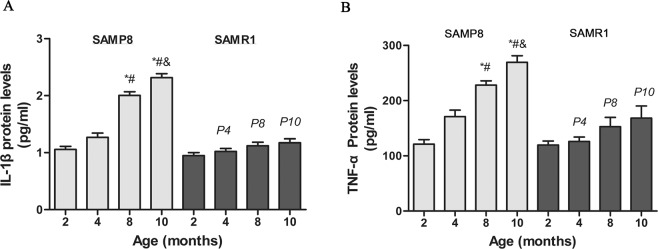
Age-related alterations in cytokines of P8 and R1 mice. (**A**,**B**) Levels of pro-inflammatory cytokines detected by xMAP. Values are present as means ± SD (n = 6). *P < 0.05 vs. 2-month-old P8; ^#^*P* < 0.05 vs. 4-month-old P8; ^&^*P* < 0.05 vs. 8-month-old P8; a *P* < 0.05 vs. 2-month-old R1; b *P* < 0.05 vs. 4-month-old R1; c *P* < 0.05 vs. 8-month-old R1; p4 *P* < 0.05 vs. 4-month-old P8; p8 *P* < 0.05 vs. 8-month-old P8; p10 *P* < 0.05 vs. 10-month-old P8.

A two-way ANOVA was used to test the values of IL-1β and TNF-α in the two strains (P8, RI) by age (2, 4, 8, 10 months old) (Fig. 3A,B). The result showed that the interaction of strain and age was not significant. In both strains of mice at 8 months old, increases in the levels of TNF-α and IL-1β were observed (*P* < 0.001) (Fig. 3A,B). Differences in the levels of IL-1β and TNF-α between the strains were detected at 4, 8 and 10 months (*P* < 0.001). Thus, pro-inflammatory cytokines TNF-α and IL-1β showed age-related elevation, and the cells were in a chronic inflammatory state.

### Age-related increase in COX2 expression in testis samples

**Figure 4:**
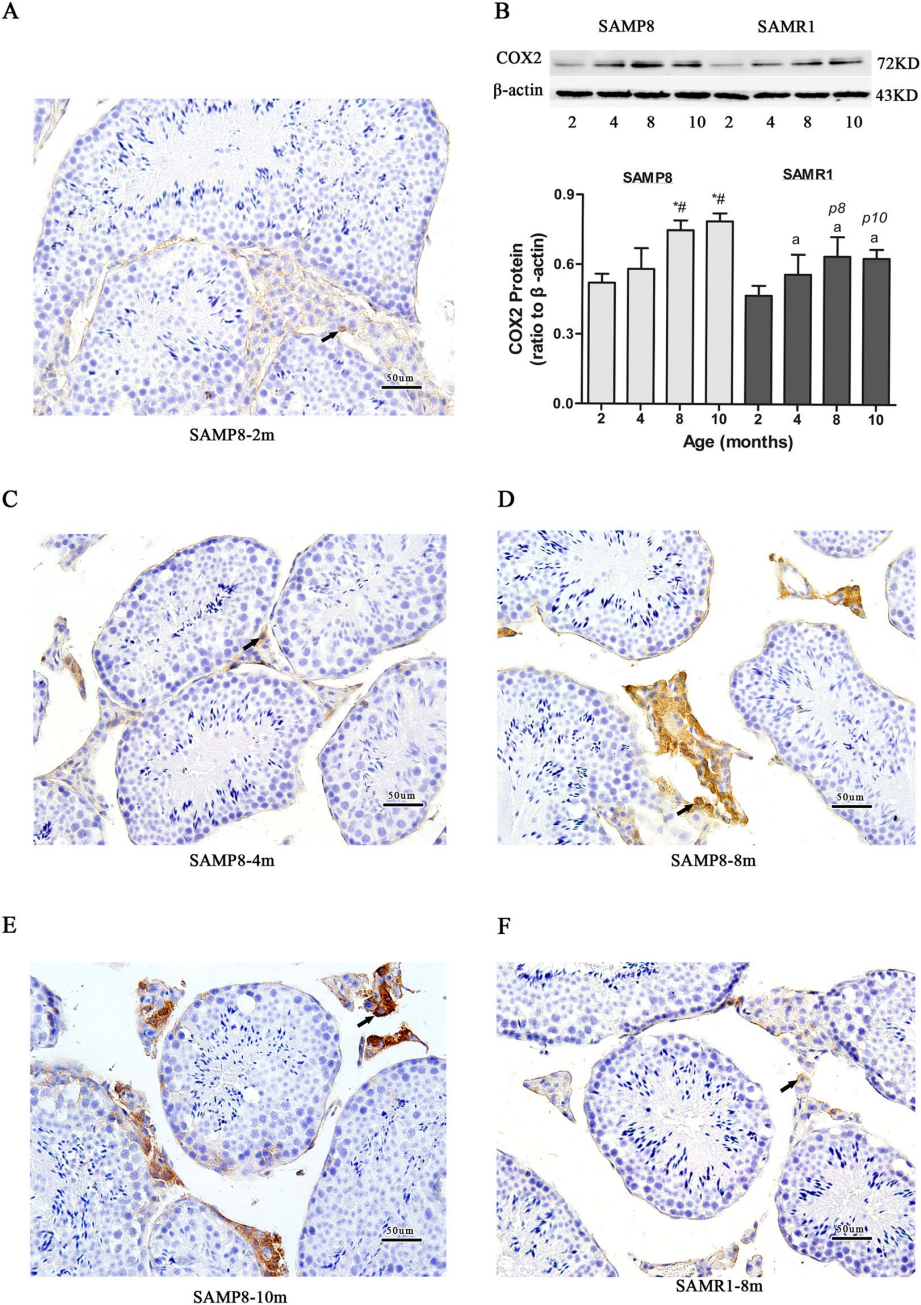
COX2 expression in P8 and R1 mice of varying ages. Testes sections were examined from the P8 groups at 2, 4, 8 and 10 months and the R1 group at 8 months of age. (**A**,**C**–**F**) COX2 protein expression by immunohistochemistry (×200, Bar:20 um). The arrow show positive Leydig cells in the interstitial tissue. (**B**) COX2 protein expression by Western blot. The expression of COX2 are based on normalized ratios to β-actin. Values are present as means ± SD (n = 6). *P < 0.05 vs. 2-month-old P8; ^#^*P* < 0.05 vs. 4-month-old P8; ^&^*P* < 0.05 vs. 8-month-old P8; a *P* < 0.05 vs. 2-month-old R1; b *P* < 0.05 vs. 4-month-old R1; c *P* < 0.05 vs. 8-month-old R1; p2 *P* < 0.05 vs. 2-month-old P8; p4 *P* < 0.05 vs. 4-month-old P8; p8 *P* < 0.05 vs. 8-month-old P8; p10 *P* < 0.05 vs. 10-month-old P8.

The levels of COX2 were tested using a two-way ANOVA in the two strains (P8, RI) by age (2, 4, 8, 10 months old) (Fig. 4A–F). The result showed that the interaction of age and strain had no differences. The protein level of COX2 in testes increased in an age-related manner in P8 and R1 mice. The level of COX2 increased at 8 and 10 months compared with that of 2 and 4 months in P8 mice. The level of COX2 in P8 became significantly higher at 8 months old than that of age-matching R1 mice (*P* < 0.05) (Fig. 4B).

A significant increment of COX2 protein level was observed in the testes of 8 months old P8 mice (Fig. 4B). Furthermore, we assessed the COX2 protein level by immunohistochemical staining, which revealed COX2 is located in the cytoplasm of interstitial cells, and the intensity of positive products elevated with aging in P8 mice (Fig. 4A–E). In P8 mice, the COX2 immunohistochemical staining was much more intense than that in R1 mice at 8 months old (Fig. 4D,F).

### Effect of age on testosterone production from isolated Leydig cells

**Figure 5:**
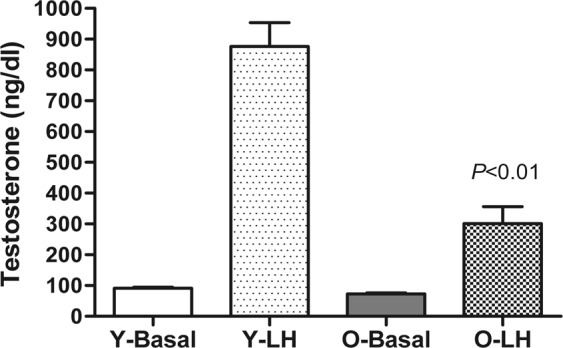
Testosterone production by isolated Leydig cells from young and old mice at basel and LH stimulation. Leydig cell suspensions were prepared from young (4 months old) and old (8–10 months old) animals, and suitable aliquots (5 × 10^5^ cells) were treated with or without LH (100 ng/ml) for 2 h at 37 °C. The amount of testosterone produced was detected by RIA. *P* < 0.01 vs. Y-LH. The data are present as means ± SD from four separate experiments.

The young Leydig cells stimulated by the maximum dose of LH showed a significantly increase in testosterone production compared to basal levels. However, the level of testosterone in old Leydig cells stimulated by the maximum dose of LH was significantly lower than that of young Leydig cells (Fig. 5).

### Effect of age on ROS production of isolated Leydig cells

**Figure 6:**
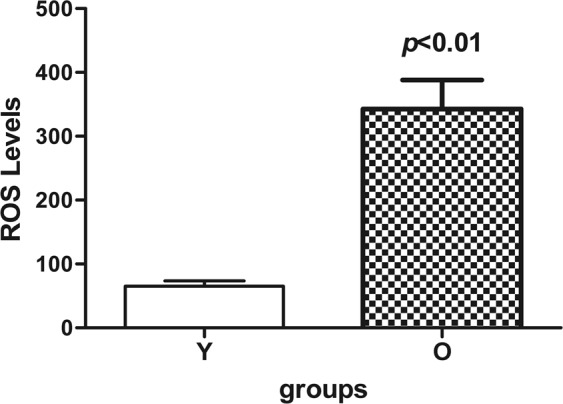
Cellular ROS production in response to advancing age was detected by flow cytometry analysis of Leydig cells. The data are present as means ± SD from four separate experiments.

Levels of ROS were significantly increased in old Leydig cells compared to the young Leydig cells (*P* < 0.05) (Fig. 6). The results indicate that aging significantly increases the intracellular ROS production, and excess ROS production may cause oxidative damage to old Leydig cells.

### Effects of p38MAPK → COX2 and NF-κB → COX2 signaling pathways on testosterone reduction of aging Leydig cells

#### Inhibition of Bay 11-7082 on NF-κB translocation to nucleus and inhibition of SB203580 on p38 MAPK

**Figure 7:**
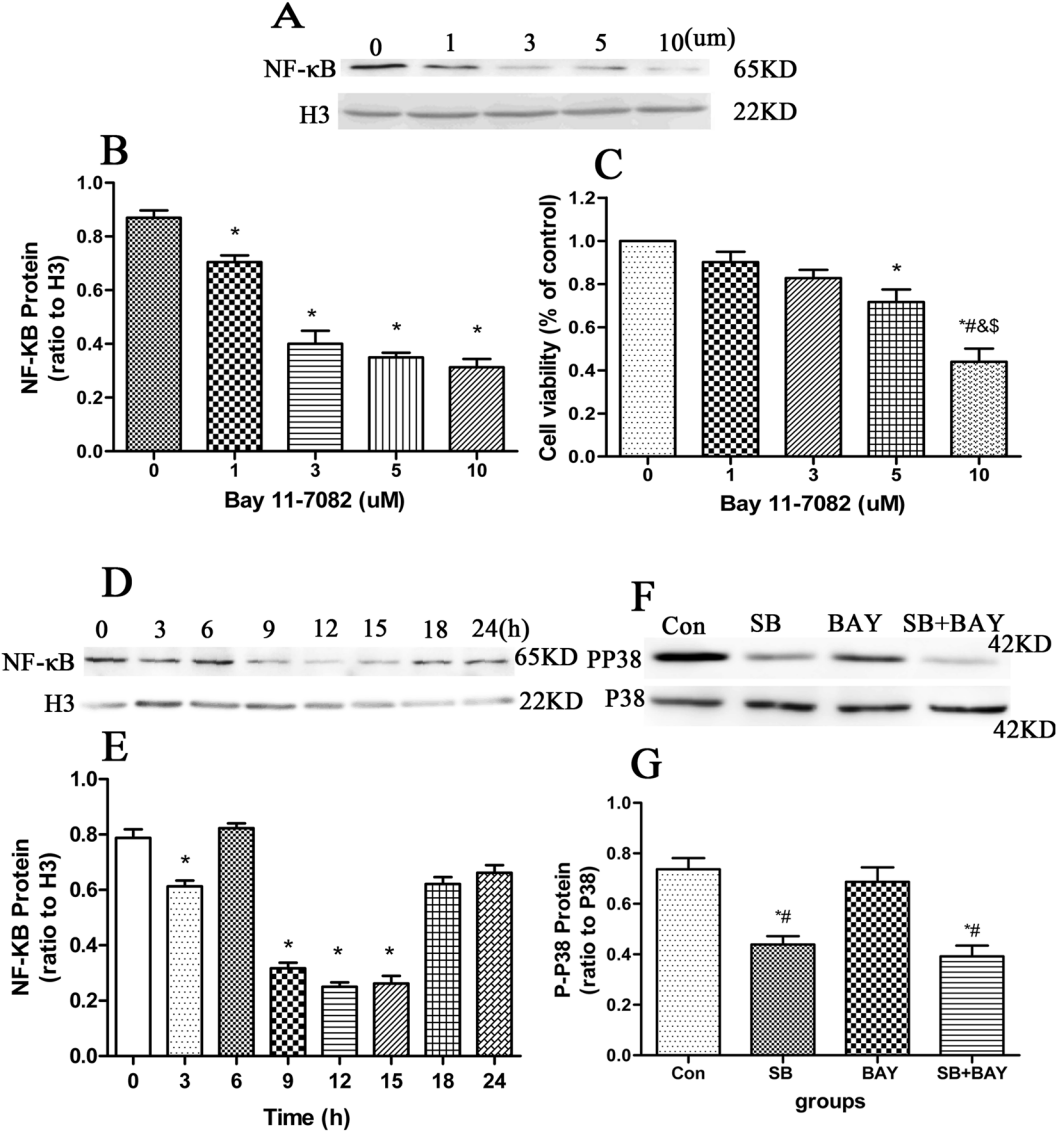
Inhibitory effects of Bay 11-7082 and SB203580 on NF-κB transcriptional activity and p38 MAPK in young and old mice Leydig cells. (**A**,**B**) Effect of Bay 11-7082 at indicated concentrations for 12 h on inhibition of NF-κB protein expression was examined by Western blot. *P < 0.05 vs. con group. (**C**) Cell viability with Bay 11-7082 at indicated concentrations for 12 h was detected by MTT assay. *P < 0.05 vs. con group; ^#^*P* < 0.05 vs. 1 µM Bay; ^&^*P* < 0.05 vs. 3 µM Bay; ^$^*P* < 0.05 vs. 5 µM Bay. (**D**,**E**) Effect of 5 μM Bay 11-7082 for indicated time on NF-κB protein expression was examined by Western blot. *P < 0.05 vs. con group. (**F**,**G**) Effect of inhibition of SB203580 on p38 MAPK was detected by Western blot. Relative P-P38 expression are present as ratios to P38. Leydig cells were treated with SB203580 at a dose of 10 µM for 1 h. *P < 0.05 vs. con group; ^#^*P* < 0.05 vs. Bay group.

We assessed the effect of Bay 11-7082 at different concentrations and different times on the inhibition of NF-κB activity using western blot and MTT assays, as well as the role of SB203580 in inhibiting p38 MAPK. Old Leydig cells were incubated with Bay 11-7082 at a doses of 0–10 μM for 12 h. As shown in Fig. 7A, the expression of *NF-κB* decreased in a dose-depentent manner when treated with Bay 11-7082. The MTT assay showed that 5 μM Bay 11-7082 treatment resulted in a 25% reduction in cell viability, but dose of 10 μM led to a 60% decrease (Fig. 7B). Bay 11-7082 treatment of old Leydig cells at the 5 μM concentration seems to be more appropriate for this study. Therefore, Leydig cells were treated with 5 μM Bay 11-7082 for the specified time (Fig. 7C). The decrease in NF-κB protein was first detected at 9 h, reached the lowest level at 12 h, then progressively increased in the next 12 h. Old Leydig cells were incubated with SB203580 (10 µM) for 1 h based on a previously published protocol^10^.

#### Effects of NF-κB and p38 MAPK inhibitor on testosterone production of young and old Leydig cells

**Figure 8:**
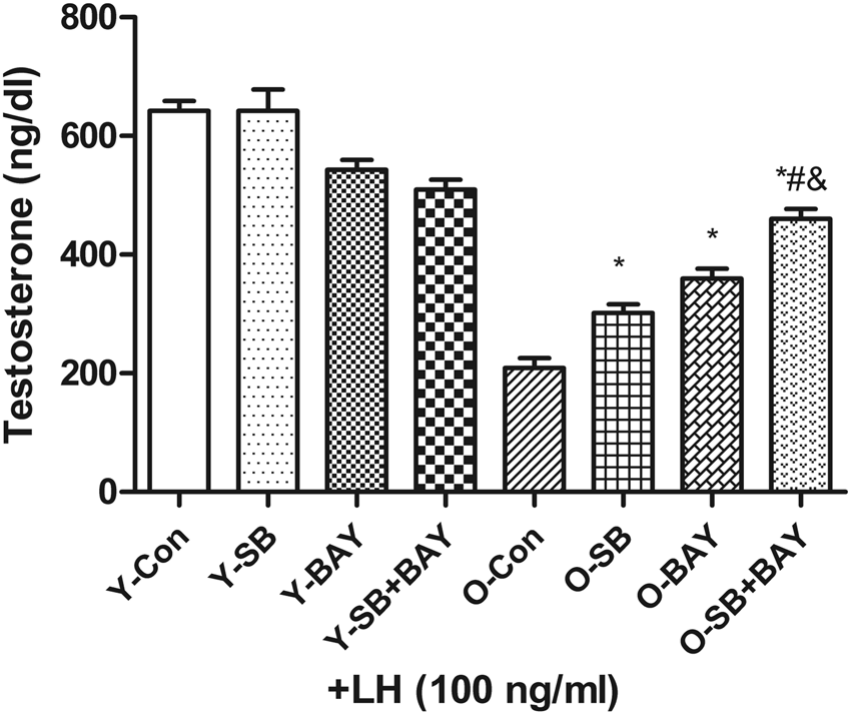
Effects of p38 MAPK signaling pathway inhibitor and NF-κB inhibitor on testosterone production stimulated by LH in Leydig cells isolated from young and old mice. Freshly isolated Leydig cells from young mature (4 months old) or old (8–10 months old) animals were treated for 2 h in culture medium supplemented with +LH (100 ng/ml) ± SB203580 (10 µM), ±Bay 11-7082 (5 μM). After treatment, the testosterone production in medium samples were quantitatively analyzed by RIA. The data are present as means ± SD from four separate experiments.*P < 0.05 vs. O-con group; ^#^*P* < 0.05 vs. O-SB group; ^&^*P* < 0.05 vs. O-BAY group.

The roles of p38 MAPK and NF-κB activity in regulating testosterone production during aging were analyzed using SB203580 and Bay 11-7082. The values were analyzed by two-way ANOVA. The results showed that SB203580 and Bay 11-7082 didn’t influence the testosterone production of Leydig cells from young mice(4 months old) (Fig. 8).

NF-κB activity increases with age, and previous data have shown that p38 MAPK pathway is closely related to NF-κB^22^. Therefore, we explored the potential relationship between p38 MAPK, NF-κB and age-related decrease in testosterone production by assessing the separated and combined effects of p38 MAPK and NF-κB inhibitors. As shown in Fig. 8, testosterone production in old Leydig cells pretreated with the p38 MAPK inhibitor SB203580 restored to approximately 40% of the level seen in young Leydig cells (P < 0.05). Likewise, using Bay 11-7082 partially restored testosterone level in old Leydig cells (*P* < 0.05) (Fig. 8). There are interactions between these two factors (*P* < 0.05). Therefore, our results showed that SB203580 and Bay 11-7082 exerted their respective effects and also produced synergistic effects on testosterone production in old Leydig cells.

#### Effects of SB203580 and/or Bay 11-7082 on the *StAR* and *COX2* expression of aged Leydig cells

**Figure 9:**
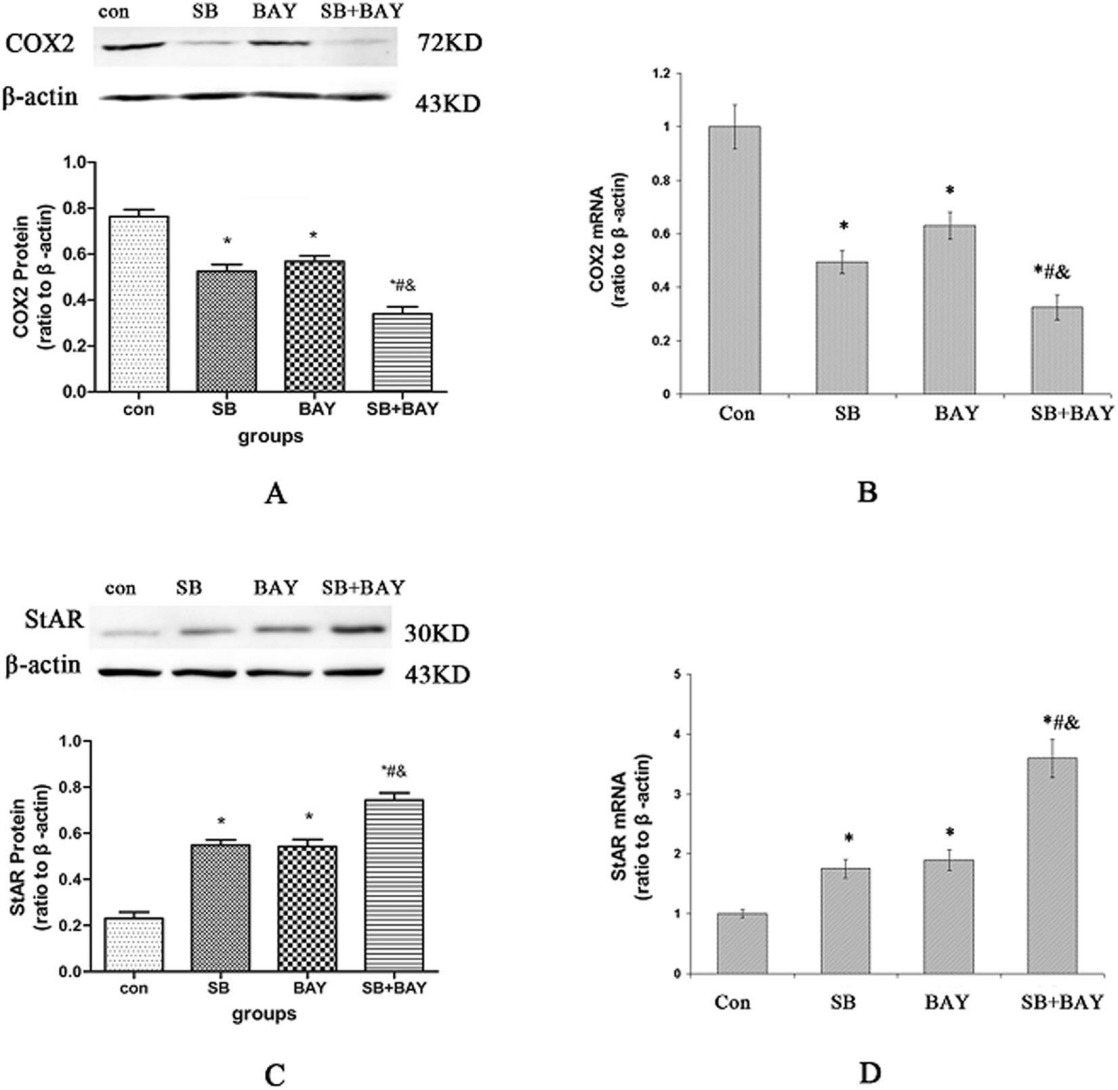
Effects of p38 MAPK inhibitor and NF-κB inhibitor on *StAR* and *COX2* gene expression in aged P8 (8–10 months old) Leydig cells. (**A**,**C**) Effects of p38 MAPK inhibitor and NF-κB inhibitor on COX2 and StAR expression were analyzed by Western blot. (**B**,**D**) Effects of p38 MAPK inhibitor and NF-κB inhibitor on *COX2* and *StAR* mRNA expression were examined by RT-PCR. The data are present as means ± SD from four separate experiments. *P < 0.05 vs. con group; ^#^*P* < 0.05 vs. SB group; ^&^*P* < 0.05 vs. Bay group.

The roles of SB203580 and Bay 11-7082 in the induction of *COX2* and *StAR* gene expression were assessed by western blotting and real-time PCR. The values were analyzed by two-way ANOVA. Bay 11-7082 or SB203580 led to a significant decline in mRNA and protein level of COX2(*P* < 0.05) (Fig. 9A,B), and a significant increase in protein and mRNA level of StAR(*P* < 0.05) (Fig. 9C,D). There are interactions between these two factors (*P* < 0.05). Treatment of cells with SB203580 and Bay 11-7082 produced a synergistic effect on *Cox2* and *StAR* gene expression. Therefore, both the NF-κB-COX2 and p38 MAPK-COX2 pathways played important roles in the reduction of testosterone production with aging.

## Discussion

Senescence has been hypothesized to be the result of accumulated oxidative injury and chronic inflammatory damage. The SAMP mice strains provide a useful tool to explore the mechanism of age-associated diseases. In comparison with the SAMR strains, the SAMP strains display an earlier onset and more accelerated progression of age-related pathological phenotypes resembling human geriatric diseases^21^. SAMP8 were used in our study to investigate serum LH across P8 mice of 2, 4, and 8 months of age yielded age-related increases but no significant effect, and decline in testosterone production and its possible mechanism. Our results are consistent with those reported in the literature^23^.

The current study was the first to show significant age-related increases in pro-inflammatory cytokines IL-1β, TNF-α and COX2, as well as decreases in anti-inflammatory cytokines in testis and elevation of intracellular ROS level in isolated Leydig cells of P8 mice, to indicate an increased inflammatory and oxidative stress level in the testis of P8 mice compared with R1 mice. Previous studies indicated that an elevated oxidative and inflammatory level was found in other organs, including the skin and brain, in the P8 mice compared with R1 mice, and an alteration in the cellular redox condition resulted in damage to DNA, protein and/or lipid components and ultimately caused cellular functional impairment^24,25^. In addition, we found that as ROS and inflammation levels increased during aging, steroidogenic enzymes (StAR and P450scc) reduced and led to the decline of testosterone production eventually. These observations suggested that excessive ROS generation and high levels of pro-inflammatory cytokines may impair Leydig cells function, leading to decreased steroidogenesis.

NF-κB, an important transcription factor, is involved in the regulation of expression of genes encoding pro-inflammatory molecules such as TNF-α, IL-1β, iNOS and COX2 in aging^3,26^. Since the increase of NF-κB activity have been reported in brain, myocardium, and liver in aged rats, NF-κB activation and chronic inflammation appear to be a common phenomenon in the aging process^15,16^. In our study, elevation of NF-κB activity was also founded in aged P8 testes. With NF-κB activation, we detected the increase in the levels of COX2, TNF-α and IL-1β in testes of 8-month-old P8 mice, which indicates that aging cells are in a chronic inflammatory condition. Among these cytokines, COX2 has been widely researched in testis. Studies have reported that inhibition of Leydig cell steroidogenesis and StAR protein expression is COX2-dependent^18^. Our results indicated that COX2 protein increased, and simultaneously StAR protein and blood testosterone level reduced in mice testes from 2 months to 10 months. NF-κB activation and chronic inflammation in the testes may be associated with the age-related COX2 elevation and testicular dysfunction in P8 mice. One possible explanation of how activation of NF-κB might inhibit steroidogenesis could be through COX2 activation. To test this hypothesis, Bay 11-7082, a specific inhibitor of NF-kB, was utilized to inhibit the activity of NF-kB in old Leydig cells. The results confirmed our hypothesis that treatment with Bay 11-7082 down-regulated the *COX2* gene expression and up-regulated the *StAR* gene expression, and then partially restored testosterone secretion in old Leydig cells. This report is the first to implicate that the inflammation-related NF-kB → COX2 signaling cascade acts as a negative regulator of testosterone production.

p38 MAPK signaling has a well-documented link to the NF-κB pathway^27^. We found activation of p38 MAPK in 8-month-old P8 mice. It is known that the activation of p38 MAPK depended on ROS directly modulates COX2 expression^28,29^. Thus, we speculate that COX2 involvement in inhibition of testosterone production may be mediated by p38 MAPK. This hypothesis is in coincidence with our results that p38 MAPK inhibitor SB203580 was shown to down-regulate *COX2* gene expression and up-regulate *StAR* gene expression, along with the increase of testosterone production in old Leydig cells. Our experiments show evidence that the higher expression of *COX2* and its negative regulation on testosterone production are mediated by pathway of p38 MAPK in old Leydig cells. Our results delineated the mechanism by which oxidative stress-associated pathway of p38 MAPK → COX2 inhibits testosterone production.

The potential relationship between p38 MAPK → COX2 and NF-kB → COX2 pathways were further explored. Simultaneous addition of SB203580 and Bay 11-7082 yielded an additive or synergistic effect on up-regulation of StAR expression and testosterone secretion in old Leydig cells. Perhaps both of the two signaling pathways, p38 MAPK → COX2 and NF-κB → COX2, play negative and synergistic regulatory roles in testosterone production with aging.

## Summary and Conclusions

Summary diagram shows a model of old Leydig cells reported in this paper. our results supply direct evidence that oxidative stress and chronic inflammation are implicated in the negative regulation of testosterone production both *in vivo* and *in vitro* in the process of aging. Furthermore, we provide evidence that activation of both p38 MAPK → COX2 and NF-κB → COX2 signaling pathways are functionally linked to the oxidative stress response and chronic inflammation during aging and mediates their inhibitory effects on testosterone production.

## Supplementary information

supplementary info including full length western blot
