# Author Correction: The roles of p38 MAPK → COX2 and NF-κB → COX2 signal pathways in age-related testosterone reduction

**DOI:** 10.1038/s41598-020-61687-8

**Published:** 2020-03-10

**Authors:** Yu Zhao, Xuehui Liu, Yine Qu, Lixuan Wang, Dan Geng, Wei Chen, Li Li, Yangyang Tian, Shiyang Chang, Chunfang Zhao, Xiujun Zhao, Pin Lv

**Affiliations:** 1 Department of Histology and Embryology, Hebei Medical University, Shijiazhuang, 050017 China; 2 Department of Occupational and Environmental Health, Hebei Province Key Laboratory of Environment and Human Health, Hebei Medical University, Shijiazhuang, 050017 China; 3 Department of Histology and Embryology, School of Basic Medical Sciences, North China University of Science and Technology, 063210 Hebei Province, China; 4 Department of human anatomy, Hebei Medical University, Shijiazhuang, 050017 China; 5 Department of cell biology, Hebei Medical University, Shijiazhuang, 050017 China

Correction to: *Scientific Reports* 10.1038/s41598-019-46794-5, published online 22 July 2019

The original version of this Article contained errors in Affiliations 3 and 4, which were incorrectly given as ‘Department of Occupational and Environmental Health, Hebei Key Laboratory of Environment and Human Health, Hebei Medical University, Shijiazhuang, 050017, China’ and ‘Department of Histology and Embryology, Medical Faculty, Hebei Union University, 063000, Hebei Province, China’ respectively.

The correct affiliations are listed below.

Affiliation 3:

Department of Occupational and Environmental Health, Hebei Province Key Laboratory of Environment and Human Health, Hebei Medical University, Shijiazhuang 050017, China.

Affiliation 4:

Department of Histology and Embryology, School of Basic Medical Sciences, North China University of Science and Technology, 063210, Hebei Province, China.

Furthermore, in the original version of this Article, Affiliations 2, 3, 4 and 5 were not listed in the correct order. The correct affiliations are listed below.

Affiliation 2:

Department of Occupational and Environmental Health, Hebei Province Key Laboratory of Environment and Human Health, Hebei Medical University, Shijiazhuang 050017, China.

Affiliation 3:

Department of Histology and Embryology, School of Basic Medical Sciences, North China University of Science and Technology, 063210, Hebei Province, China.

Affiliation 4:

Department of human anatomy, Hebei Medical University, Shijiazhuang 050017, China.

Affiliation 5:

Department of cell biology, Hebei Medical University, Shijiazhuang 050017, China.

These errors have now been corrected in the PDF and HTML versions of the Article, and in the accompanying Supplementary information.

