# Supplementary material for: The roles of p38 MAPK → COX2 and NF-κB → COX2 signal pathways in age-related testosterone reduction
Source: Sci Rep. 2019 Jul 22;9:10556. doi: 10.1038/s41598-019-46794-5 (PMC6646396; doi:10.1038/s41598-019-46794-5)
Supplement: Supplementary file 1 — supplementary info including full length western blot [file 41598_2019_46794_MOESM1_ESM.docx]

**Title page:**

**Title: The roles of p38 MAPK→COX2 and NF-κB→COX2 signal pathways in**

**age-related testosterone reduction**

**Author list:**

1. Yu Zhao, Department of Histology and Embryology, Hebei Medical University, 361 Eastern Zhongshan Road, Shijiazhuang 050017, China.
2. Xuehui Liu, Department of Occupational and Environmental Health, Hebei Province Key Laboratory of Environment and Human Health, Hebei Medical University, Shijiazhuang 050017, China
3. Yine Qu, Department of Histology and Embryology, School of Basic Medical

Sciences, North China University of Science and Technology, 063210, Hebei

Province, China.

1. Lixuan Wang, Department of Histology and Embryology, Hebei Medical University, 361 Eastern Zhongshan Road, Shijiazhuang 050017, China.
2. Dan Geng, Department of human anatomy, Hebei Medical University, 361 Eastern Zhongshan Road, Shijiazhuang 050017, China.
3. Wei Chen, Department of Histology and Embryology, Hebei Medical University, 361 Eastern Zhongshan Road, Shijiazhuang 050017, China.
4. Li Li，Department of Histology and Embryology, Hebei Medical University, 361 Eastern Zhongshan Road, Shijiazhuang 050017, China.
5. Yangyang Tian, Department of Histology and Embryology, Hebei Medical University, 361 Eastern Zhongshan Road, Shijiazhuang 050017, China.
6. Shiyang Chang, Department of Histology and Embryology, Hebei Medical University, 361 Eastern Zhongshan Road, Shijiazhuang 050017, China.
7. Chunfang Zhao, Department of Histology and Embryology, Hebei Medical University, 361 Eastern Zhongshan Road, Shijiazhuang 050017, China.
8. Xiujun zhao*****, Department of Histology and Embryology, Hebei Medical

University, 361 Eastern Zhongshan Road, Shijiazhuang 050017, China

1. Pin Lv*****, Department of cell biology, Hebei  Medical University, Shijiazhuang

050017, China

***Corresponding author:**

1, Xiujun Zhao, Ph.D. Associated Professor. Department of Histology and Embryology, Hebei Medical University, Shijiazhuang，050017, China.

2, Pin lv, Ph.D. Professor. Department of cell biology, Hebei  Medical University,

Shijiazhuang 050017, China

Tel: 86-311-86266082

Fax: 86-311-86043026

E-mail : [xiujunzhao1977@126.com](mailto:xiujunzhao1977@126.com)

**Figures**

**Figure.1**


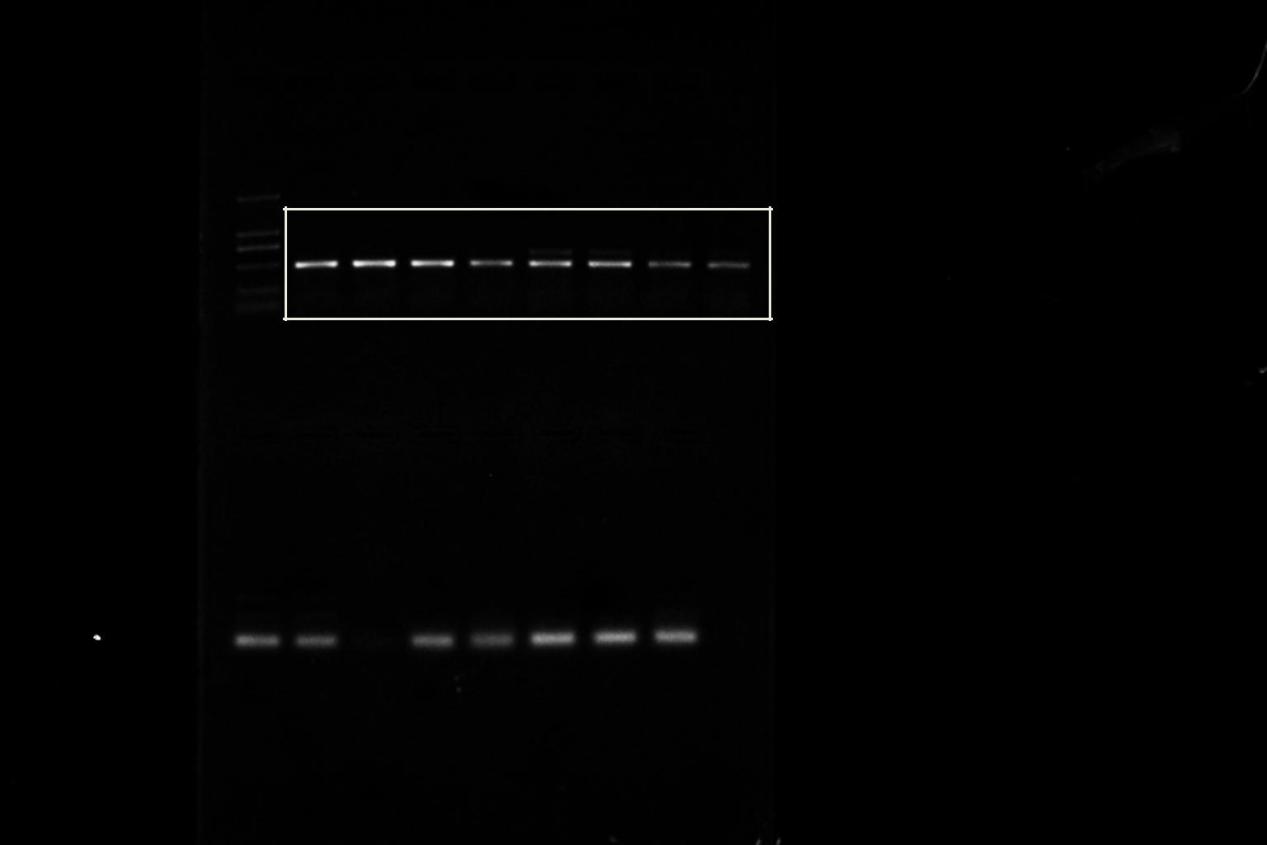


**Figure.1.** Expressions of lenses P450scc by PCR assays. Full-length blots of figure

2(A)

**Figure.2**


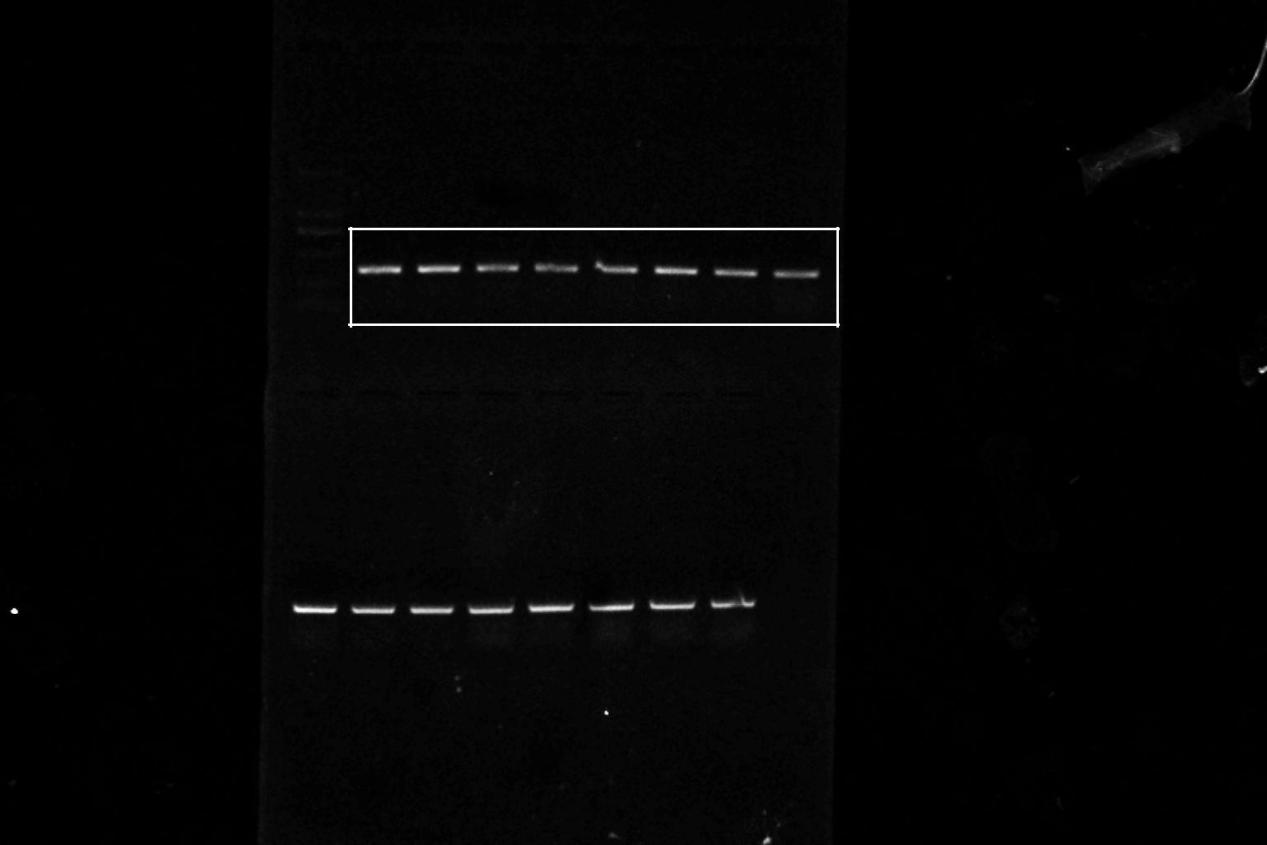


**Figure.2.** Expressions of lense StAR by PCR assays. Full-length blots of figure 2(B)

**Figure.3.**


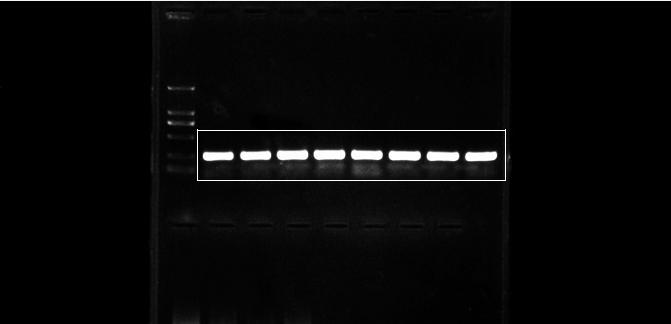


**Figure.3.** Expressions of lense β-actin by PCR assays. Full-length blots of figure 2(B)

**Figure.4.**


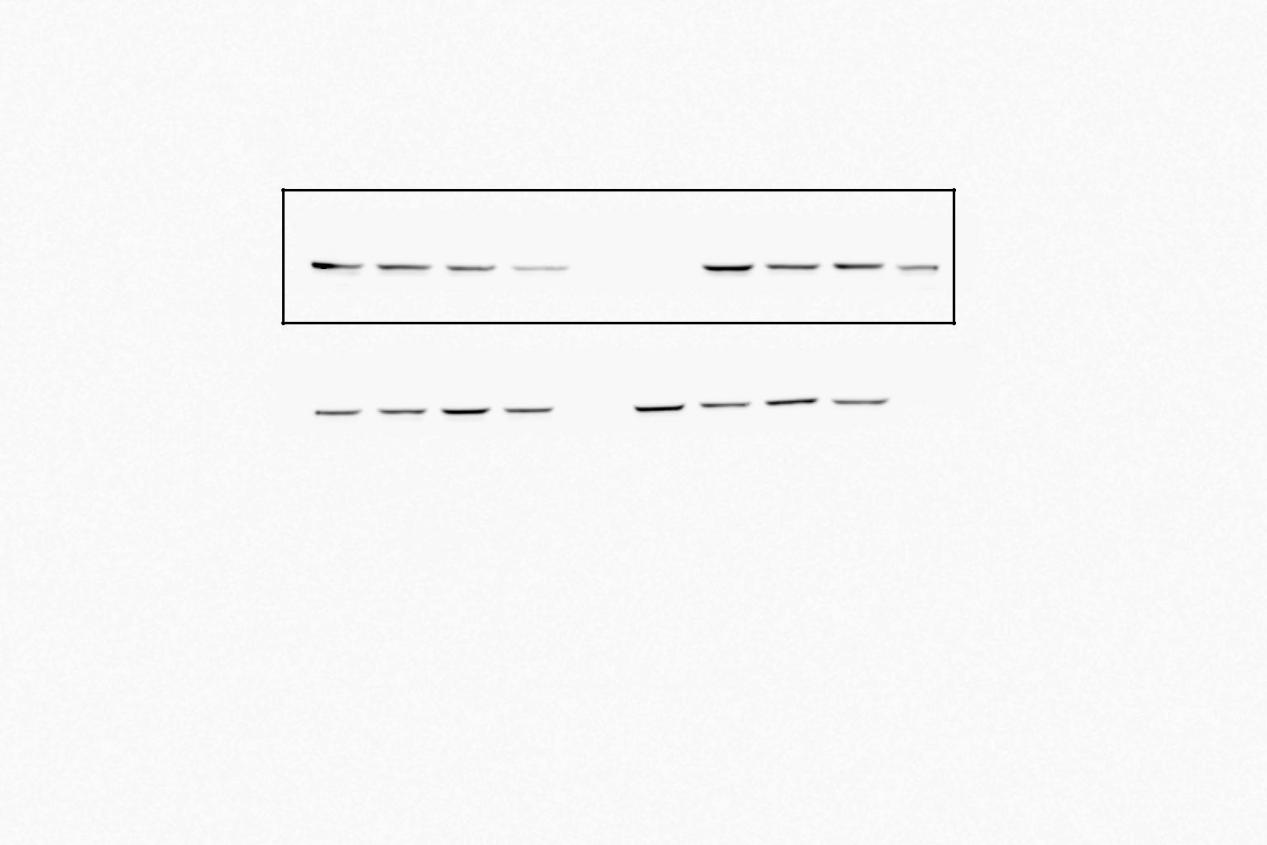


**Figure.4.** Expressions of lenses P450scc by Western blot assays. Full-length blots of figure2(C).

**Figure.5.**


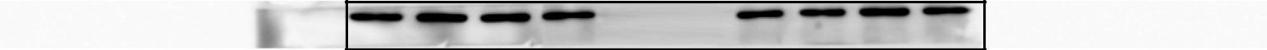


**Figure.5.** Expressions of lenses β-actin by Western blot assays. Full-length blots of figure2(C).

**Figure.6.**


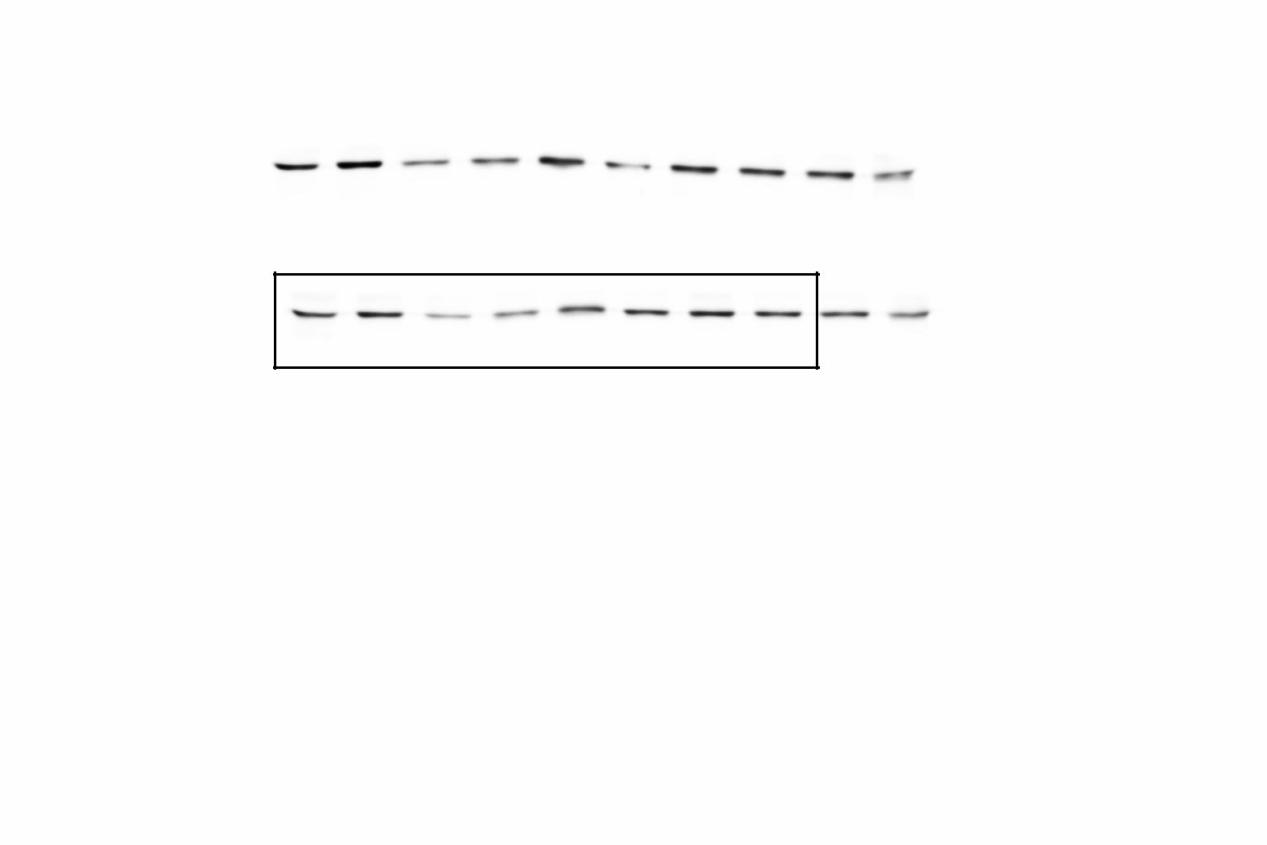


**Figure.6.** Expressions of lenses StAR by Western blot assays. Full-length blots of

figure 2(D).

**Figure.7.**


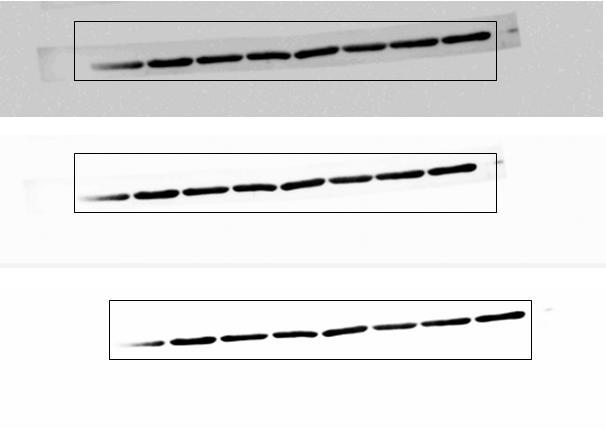


**Figure.7.**Expressions of lenses β-actin by Western blot assays at multiple exposures. Full-length blots of figure 2(D)

**Figure.8.**


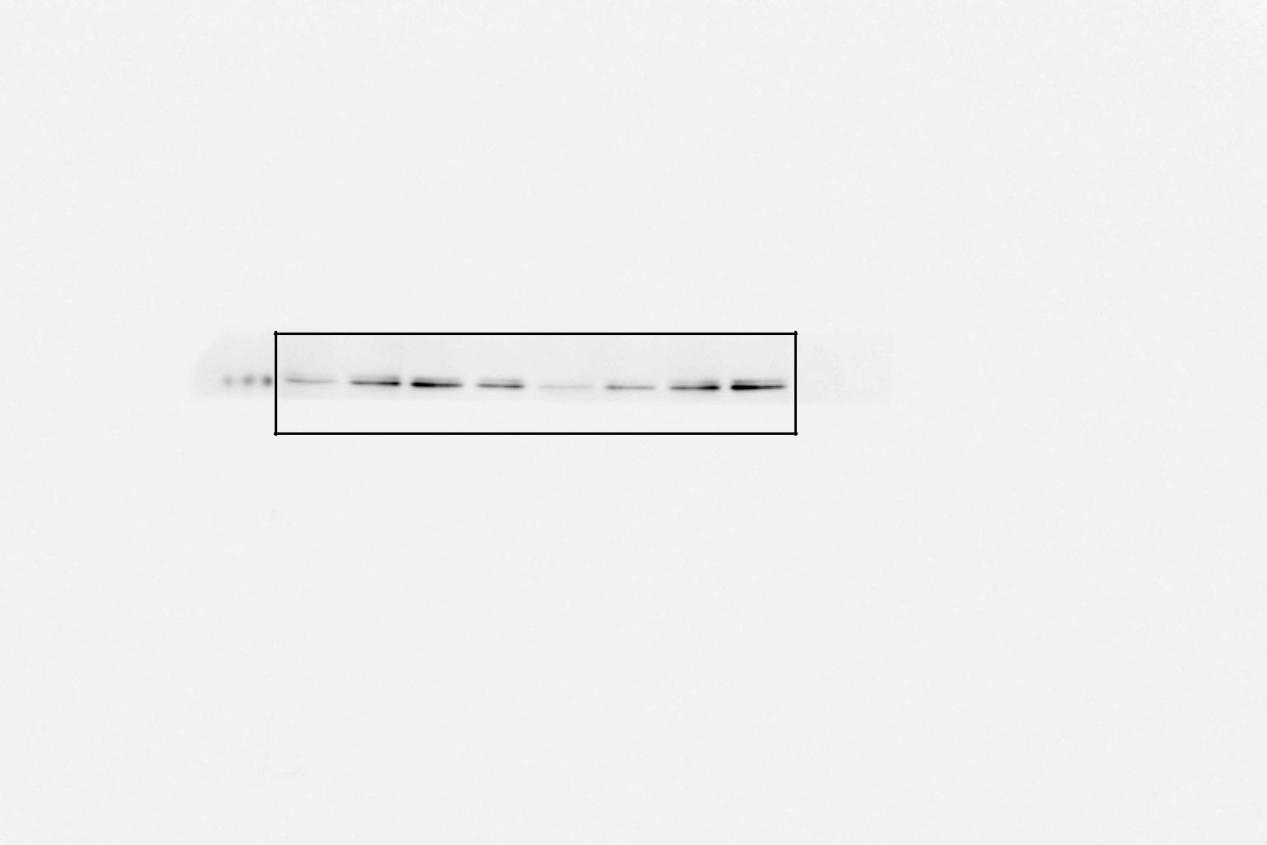


**Figure.8.** Expressions of lenses COX2 by Western blot assays. Full-length blots of figure 4(B).

**Figure.9.**


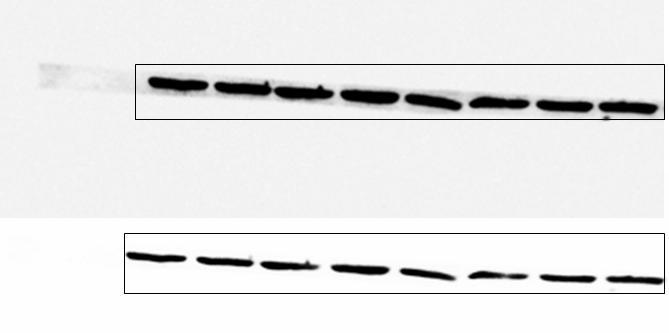


**Figure.9.**Expressions of lenses β-actin by Western blot assays at multiple exposures.

Full-length blots of figure 4(B).

**Figure.10.**


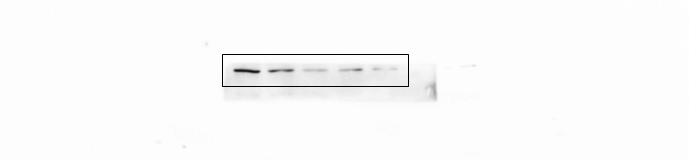


**Figure.10.** Expressions of lenses NF-κB by Western blot assays. Full-length blots of figure.7.(A).

**Figure.11.**


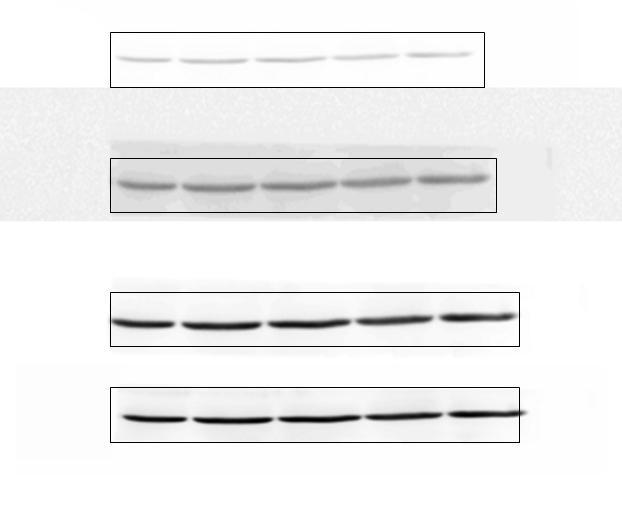


**Figure.11.**Expressions of lenses H3 by Western blot assays at multiple exposures.

Full-length blots of figure 7(A).

**Figure.12.**


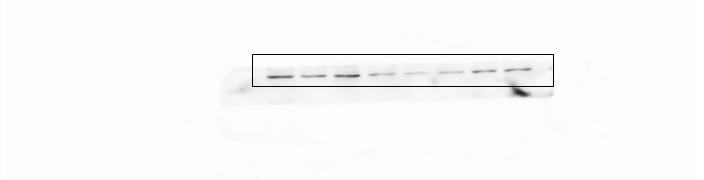


**Figure.12.** Expressions of lenses NF-κB by Western blot assays. Full-length blots of figure.7.(D).

**Figure.13.**


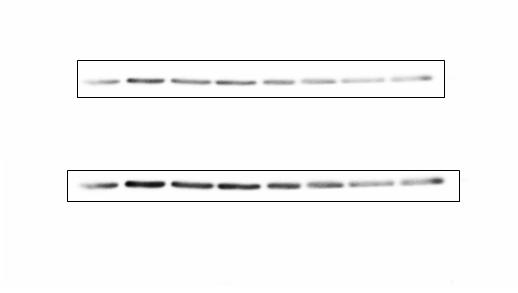


**Figure.13.** Expressions of lenses H3 by Western blot assays at multiple exposures.

Full-length blots of figure 7(D).

**Figure.14.**


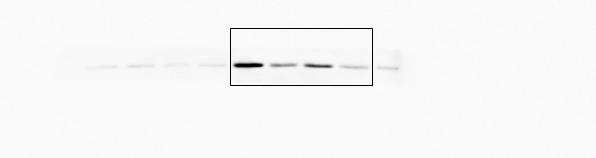


**Figure.14.** Expressions of lenses PP38 by Western blot assays. Full-length blots of figure.7.(F).

**Figure.15.**


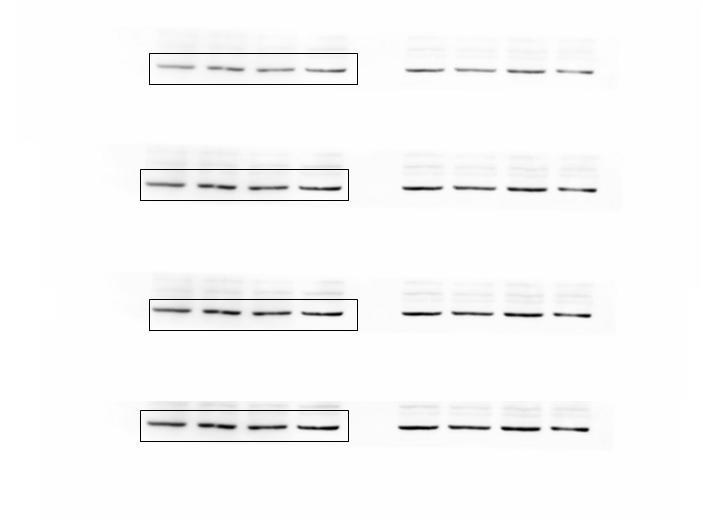


**Figure.15.**Expressions of lenses P38 by Western blot assays at multiple exposures.

Full-length blots of figure 7(F).

**Figure.16.**


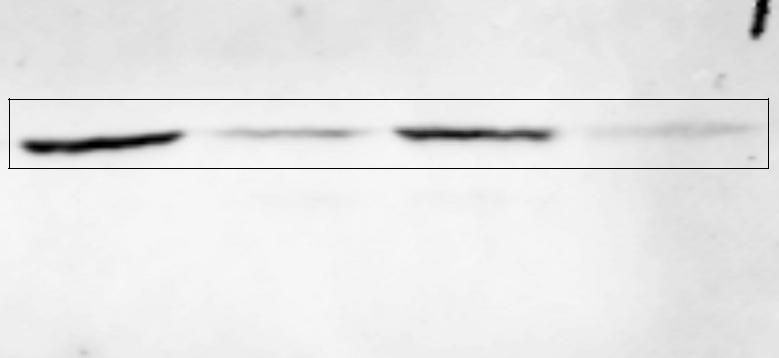


**Figure.16.** Expressions of lenses COX2 by Western blot assays. Full-length blots of figure.9.(A).

**Figure.17.**


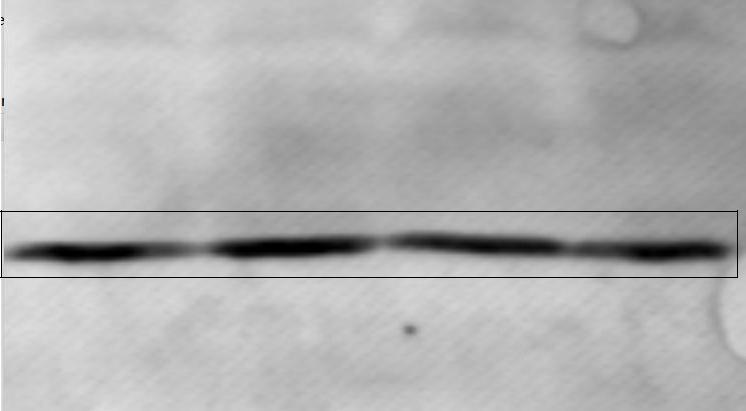


**Figure.17.**Expressions of lenses β-actin by Western blot assays at multiple exposures.

Full-length blots of figure9 (A).

**Figure.18.**


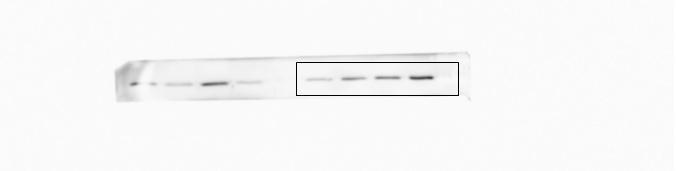


**Figure.18.** Expressions of lenses StAR by Western blot assays. Full-length blots of

figure.9.(C)

**Figure.19.**


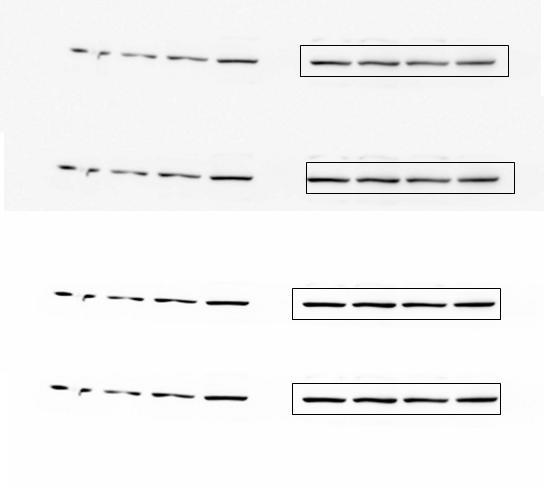


**Figure.19.**Expressions of lenses β-actin by Western blot assays at multiple exposures.

Full-length blots of figure9 (C).
